# An Improved Hippopotamus Optimization Algorithm with Trend-Aware Mechanism for Fake Information Detection

**DOI:** 10.3390/biomimetics11100697

**Published:** 2026-10-01

**Authors:** Xu Dai, Guoqiang Lu, Jiaxue Li

**Affiliations:** 1 School of Finance, Jilin University of Finance and Economics, Changchun 130117, China; daixusissi@jlufe.edu.cn; 2 Jilin Provincial Key Laboratory of Fintech, Changchun 130117, China; 3 School of Health Management, Mudanjiang Medical University, Mudanjiang 157011, China; luguoqiang_123@163.com; 4 Institute of National Development and Security Studies, Jilin University, Changchun 130012, China

**Keywords:** fake information detection, hippopotamus optimization algorithm, trend-aware mechanism, chaotic mapping, dual-channel

## Abstract

Fake information detection remains challenging because deceptive texts often contain complex semantic structures, while time-consuming hyperparameter tuning limits model performance. To address these issues, this study proposes IHOA-CNN-BiLSTM. This hybrid detection framework integrates the Improved Hippopotamus Optimization Algorithm (IHOA) with an optimized parallel dual-channel deep learning architecture to enhance detection accuracy. Firstly, the Logistic-Tent hybrid chaotic mapping replaces random population initialization, improving coverage uniformity and ergodicity in the search space. Secondly, the trend-aware mechanism (TAM) captures population evolution trends by maintaining historical search positions and a fitness matrix. An adaptive covariance matrix generates offset vectors that provide trend guidance during exploration, exploitation, and local optima escape stages, thereby balancing global and fine-grained local search. Thirdly, a CNN-BiLSTM parallel dual-channel feature-extraction network is designed, with key hyperparameters jointly optimized by IHOA to address fake information detection tasks. Experiments on the PolitiFact dataset show the proposed model achieves an accuracy of 90.13%, exceeding the optimal hybrid model by 2.36 percentage points. On the Weibo21 dataset, the accuracy and F1-score are 5.59 and 5.84 percentage points higher than the average values of the nine optimized CNN-BiLSTM baselines. Overall, this work presents a practical and efficient approach for detecting fake information.

## 1. Introduction

In the digital age, social network platforms have become central to the generation and dissemination of public opinion [1,2]. This decentralized mode of sharing information empowers individuals’ discourse rights, yet it substantially reduces the barrier for creating and spreading fake information [3,4]. Fake information can be packaged in news-like or otherwise credible-looking forms, while increasingly coherent machine-generated narratives make traditional surface-feature detection less effective [5]. The Digital News Report 2025, released by the Reuters Institute for the Study of Journalism at the University of Oxford, presents findings from a survey conducted across 48 markets worldwide. The report reveals that 58% of participants struggle to differentiate between true and fake news online [6]. The proliferation of fake information distorts public cognition, intensifies social divides, and affects crucial domains such as financial markets and responses to public health crises [7]. Therefore, developing artificial intelligence systems capable of automatically and accurately identifying fake information is of urgent practical significance and extensive research value.

Early fake information detection methods primarily relied on feature engineering, extracting linguistic, psychological, or propagation features such as sentiment intensity, readability indices, and propagation tree depth, and inputting them into machine learning classifiers [8,9]. However, the performance of these methods heavily depends on expert knowledge. In addition, feature designs are typically tailored to specific types of fake information, making them challenging to adapt to rapidly evolving forms [10,11]. With the rise of deep neural networks, especially pre-trained language models, fake information has entered a new stage: AI versus AI. Generative models can generate grammatically fluent, seemingly logical fake texts. Thus, detection models need to possess deeper semantic understanding and reasoning capabilities, as well as the ability to capture subtle anomalous signals from massive, high-noise datasets [12].

Subsequently, studies have focused on end-to-end deep learning models [13,14]. Among these, the hybrid architecture combining Convolutional Neural Networks (CNNs) and Bidirectional Long Short-Term Memory (BiLSTM) networks has achieved notable success [15,16]. CNNs excel at capturing local patterns, such as key phrases and abnormal collocations, through local convolutional operations. In contrast, BiLSTM effectively models long-range contextual dependencies in text sequences, thereby assessing the coherence and plausibility of the overall narrative [17,18]. This blend of local perception and global context provides an efficient framework for identifying fake information [19]. However, as with many complex neural networks, this hybrid model contains numerous hyperparameters that must be preset, such as the structure of convolutional layers, the number of recurrent layer units, and regularization hyperparameters during training [20]. These hyperparameters constitute a high-dimensional, discrete, and interactive search space, with optimal solutions varying across datasets and tasks [21]. Traditional hyperparameter search can become computationally expensive as dimensionality grows; moreover, vanilla Bayesian optimization can be difficult to scale to high-dimensional or mixed search spaces [22,23].

Metaheuristic optimization algorithms provide innovative solutions for hyperparameter optimization challenges [24]. For instance, the Hippopotamus Optimization Algorithm (HOA) is a recent population-based metaheuristic inspired by hippopotamus behaviors and implements distinct exploration, exploitation, and local optima escape mechanisms [25]. Subsequent studies have continued to benchmark and enhance HOA, indicating competitive global-search potential while also identifying limitations in diversity and convergence [26]. However, when the standard HOA is applied to the complex hyperparameter optimization of hybrid models, its inherent deficiencies become evident. First, randomness in the initial population generation strategy may lead to an uneven distribution of starting points, thereby impairing the efficiency of early-stage exploration. Second, in the later exploitation phase, population diversity declines rapidly, increasing the risk of premature convergence. Importantly, the position update formulas fail to capture the population’s overall evolutionary trend, leading to a degree of blindness in the iterative process and limiting the ability to locate the global optimum in complex landscapes.

To address the limitations of the standard HOA and enhance the automated tuning tool for detecting fake information, this paper proposes an improved Hippopotamus Optimization Algorithm (IHOA) integrated with a trend-aware mechanism (TAM). TAM is well-suited to the HOA’s multi-stage search framework because its historical search information can provide guidance across different search stages and improve the balance between exploration and exploitation. The contributions of this paper are summarized as follows:

(1) A Logistic-Tent hybrid chaotic mapping is adopted for population initialization instead of random initialization. By combining the Logistic mapping with the Tent mapping, the proposed strategy significantly improves the coverage and diversity of the initial solution space, mitigates clustering and voids caused by random generation, and thus provides a more robust starting point for subsequent global exploration.
(2) A trend-aware mechanism (TAM) is incorporated into various stages of the algorithm’s search. This mechanism continuously perceives and responds to the optimization trends emerging during the iterative process, thereby dynamically balancing exploration and exploitation and effectively improving the algorithm’s ability to escape local optima and its overall convergence performance.
(3) An automatic hyperparameter configuration framework for CNN and BiLSTM driven by the improved HOA is constructed. This framework intelligently searches for and determines the key hyperparameters of the network structure. By adaptively aligning the network architecture with the specific characteristics of fake information detection tasks, it effectively bridges the gap between model structure and task requirements, thereby fully releasing the discriminative potential of the parallel dual-channel CNN-BiLSTM architecture.

## 2. Related Work

### 2.1. Technological Evolution of Fake Information Detection

The development of fake information detection techniques has progressed from relying on explicit knowledge rules to using data-driven shallow statistical models, and now to focusing on deep neural networks [27,28]. This evolution reflects a significant shift in research emphasis from model interpretability to representation and generalization capability.

Early approaches relied heavily on expert-designed linguistic cues, deception indicators, and rule- or feature-based decision procedures [29,30]. Credibility and fact-checking systems later incorporated external evidence to assess whether claims were supported by reliable sources [31]. Such feature-driven systems can expose relatively explicit decision cues and offer greater interpretability than end-to-end black-box models [32]. However, their limitations are considerable. The construction and maintenance of rule bases require significant human effort, making them difficult to scale. Additionally, their ability to generalize rapidly declines when faced with evolving fake information that deliberately circumvents established rules.

The second stage is data-driven, combining shallow statistical models with manual feature engineering [33,34]. With increased data availability, researchers extract linguistic, user-behavioral, and simple propagation-network features from texts. They employ machine learning algorithms such as Support Vector Machines and Random Forests for classification [35,36]. Methods at this stage automated the detection process, reduced reliance on fully hand-coded rules, and achieved competitive performance on annotated datasets [37,38]. Nevertheless, the performance bottleneck primarily stems from the quality of feature representation. Handcrafted features often struggle to capture deep semantic logic and complex contextual relationships. When dealing with fake content generated by advanced technologies that closely resemble genuine information in surface features, the effectiveness of these methods is significantly compromised.

Deep neural networks dominate the third stage, achieving end-to-end feature learning and semantic understanding [39]. Deep learning models, especially Convolutional Neural Networks, Recurrent Neural Networks, and their combined hybrid architectures, can automatically learn multi-level feature representations from raw text [40,41]. By combining local feature extraction with long-range dependency modeling, they effectively enhance the model’s ability to capture complex semantic inconsistencies [42]. In recent years, pre-trained language models based on large-scale corpora have significantly improved performance [43,44]. Meanwhile, advances in generative artificial intelligence, particularly large language models, have enabled the generation of increasingly plausible misinformation, posing greater challenges to reliable detection [45,46]. Recent studies have incorporated LLM-generated reactions and explanations, as well as retrieval-augmented in-context learning, into misinformation detection to enrich contextual evidence and improve cross-domain detection performance [47,48]. However, fake news detectors can be constrained by imbalanced training data and inadequate modeling of the data distribution on target platforms [49]. Especially for hybrid and large pre-trained models, the hyperparameters of network structure and training strategies span a complex, high-dimensional space. Traditional tuning methods are inefficient, serving as a key bottleneck that limits the full release of model performance and its practical deployment. Therefore, exploring efficient, automated methods to optimize the hyperparameters of these deep learning models has become a core issue.

### 2.2. Metaheuristic Optimization Algorithms and Applications

Hyperparameter configuration is an important component of developing effective machine learning models, making systematic hyperparameter optimization necessary [50]. Conventional hyperparameter search can require substantial computational resources because numerous candidate configurations must be evaluated, thereby motivating more efficient resource allocation strategies [51]. To address this issue, metaheuristic algorithms have been widely introduced to this field due to their gradient-independent nature and global search capabilities. For instance, enhanced Grey Wolf Optimizers (GWO) have been employed to optimize CNN-LSTM architectures and learning hyperparameters, while evolutionary algorithms have also been applied to neural architecture search [52,53]. By simulating information sharing and competition among individuals in a population, these algorithms can obtain superior hyperparameter configurations compared to random search in relatively fewer iterations.

However, metaheuristic optimization methods still face challenges in dealing with high-dimensional, computationally expensive hyperparameter search spaces in machine learning and deep learning [54,55]. Firstly, the population initialization strategy affects the distribution and quality of initial solutions, thereby influencing subsequent optimization performance [56]. Secondly, insufficient exploration and loss of population diversity can lead to premature convergence to local optima [57]. Thirdly, insufficient use of historical search information can limit directional guidance during optimization; recent approaches have therefore exploited solution sequences and population trajectories to guide subsequent search updates [58].

To overcome these limitations, researchers have pursued improvements from two main perspectives. The first approach is to refine the population initialization by employing structured sampling methods, such as Latin hypercube sampling, to improve the coverage and distribution of initial solutions across the search space [59]. Another approach is to introduce cooperative multi-population mechanisms that diversify search behavior and improve the effectiveness of evolutionary optimization [60]. Recent studies have introduced auxiliary strategies with memory and foresight capabilities, enabling algorithms to adjust their step sizes and search directions by referencing historical trajectories, demonstrating promising potential for solving complex multimodal problems.

Implementing the optimization strategies mentioned above to detect fake information is clearly important in practice. Hybrid architectures combining CNNs and BiLSTMs have been applied to fake news detection to jointly capture complementary textual and sequential features [61]. However, these hybrid models involve many mutually coupled hyperparameters, whose optimal configurations vary significantly with the topic and linguistic characteristics of different datasets, making manual tuning insufficient to guarantee generalization. Metaheuristic optimization has also been incorporated into fake news detection models. For example, Whale Optimization Algorithm (WOA) has been used to optimize attention weights in an Attention-BiLSTM framework [62], while an improved Black-Winged Kite Algorithm (BKA) has been applied to optimize key hyperparameters of CNN-BiLSTM-based fake information detection models [63].

It is worth noting that the Hippopotamus Optimization Algorithm (HOA) is a recently proposed swarm-based metaheuristic that has demonstrated competitive performance across multiple benchmark test suites. Nevertheless, its application to the specific and complex task of hyperparameter tuning for fake information detection models is still in its early stages. How to integrate this algorithm with an intelligent guidance strategy that leverages iterative historical information to enhance its efficiency and stability in hybrid model hyperparameter search remains an open question that has not been thoroughly explored. Given this context, it remains important to investigate how the HOA can adopt a strategy to optimize the hyperparameters of the CNN-BiLSTM.

## 3. Methodology

**Figure 1:**
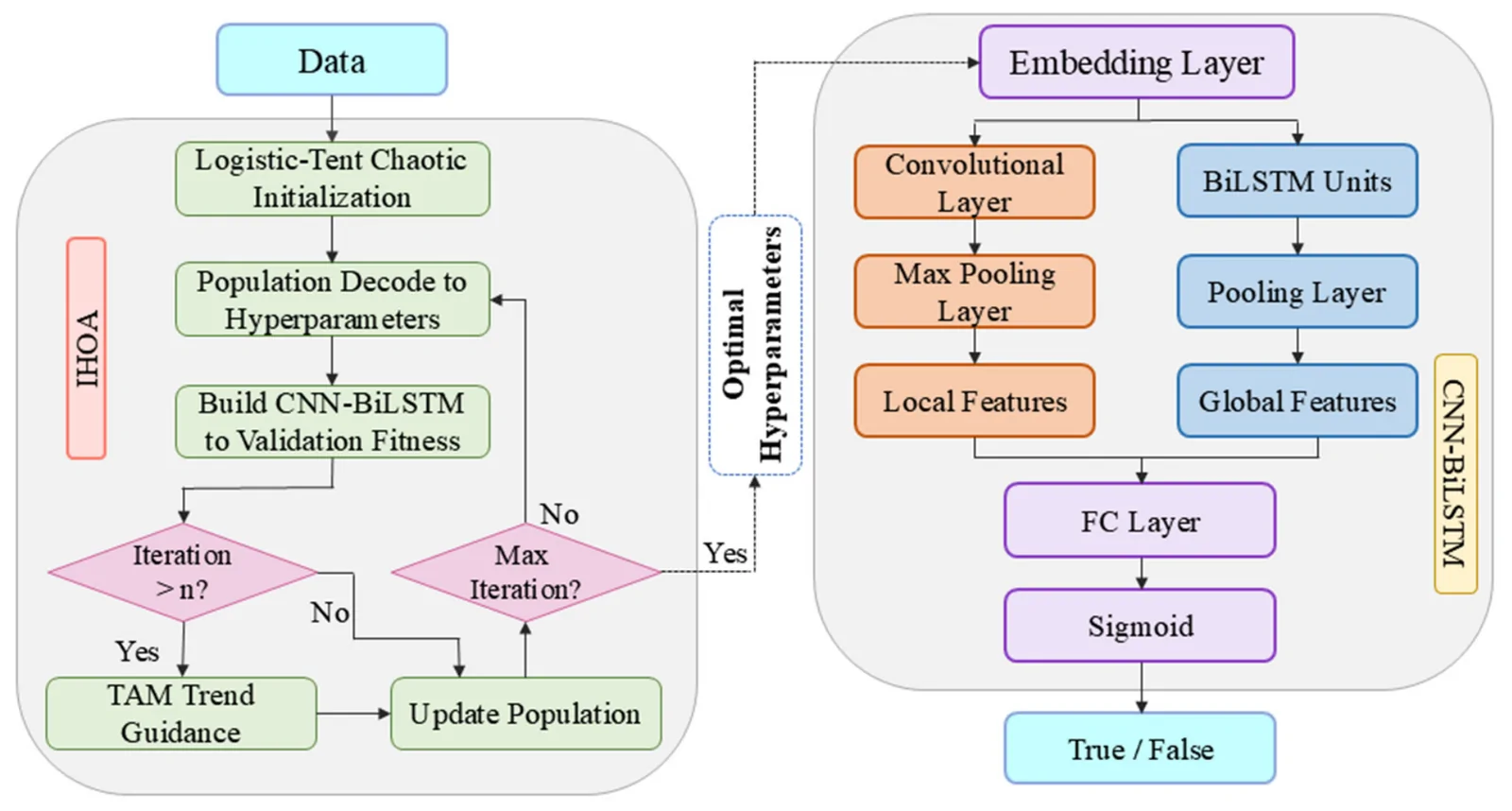
Architecture of the proposed IHOA-CNN-BiLSTM model.

Fake information on social media poses practical problems, including strong disguises, complex narrative structures, and the inability of traditional detection methods to capture its deep semantic contradictions and implicit logical fallacies. Therefore, to address these issues, this study proposes an improved Hippopotamus Optimization Algorithm that incorporates a trend-aware mechanism. First, a Logistic-Tent hybrid chaotic mapping strategy is employed to improve population initialization, thereby enhancing the uniformity and evenness of initial solution distribution in the search space. Second, a trend-aware mechanism (TAM) is introduced to dynamically monitor changes in population convergence and diversity, providing directional guidance and adaptive regulation for the algorithm’s exploration, exploitation, and local optima escape phases. Third, a parallel dual-channel automated hyperparameter optimization framework, driven by IHOA and based on CNN-BiLSTM, is constructed to achieve intelligent optimization of key hyperparameters and adaptive matching of the model structure. The overall architecture of the proposed IHOA-CNN-BiLSTM model is illustrated in Figure 1.

### 3.1. Hippopotamus Optimization Algorithm (HOA)

The Hippopotamus Optimization Algorithm (HOA) is a novel metaheuristic optimization method that simulates the social behaviors and survival strategies of hippopotamus groups [64]. The algorithm draws inspiration from three typical behaviors of hippos in their natural habitat: updating group positions in rivers or ponds, collective defense against predators, and rapid escape when facing threats. HOA transforms behaviors into mechanisms for exploration, exploitation, and local optima escape through mathematical modeling, enabling efficient global search in the solution space.

#### 3.1.1. Population Initialization

In HOA, each hippopotamus individual represents a candidate solution, and its position corresponds to the decision variables of the optimization problem. Let the population size be *N*, and the problem dimension be *m*. The positions of the entire population can be represented by matrix *X*, as shown in Equation (1).

(1) $$X=\left[\begin{array}{c} \begin{array}{c} x_{1}\\ \vdots \end{array}\\ \begin{array}{c} x_{i}\\ \vdots \end{array}\\ \begin{array}{c} x_{N} \end{array} \end{array}\right]={\left[\begin{array}{c} \begin{array}{cccc} x_{1,1} & x_{1,2} & \cdots & x_{1,m}\\ \vdots & \vdots & \ddots & \vdots \end{array}\\ \begin{array}{cccc} x_{i,1} & x_{i,2} & \cdots & x_{i,m}\\ \vdots & \vdots & \ddots & \vdots \end{array}\\ \begin{array}{cccc} x_{N,1} & x_{N,2} & \cdots & x_{N,m} \end{array} \end{array}\right]}_{N\times m}$$

Here, *X_i_* = [*x_i_*_,1_,*x_i_*_,2_,…,*x_i_*_,*m*_] denotes the position of the *i*-th hippopotamus. The initial population is generated randomly within the search space, and the initialization formula for the *j*-th dimension of the *i*-th individual is given by Equation (2).

*x_i_*_,*j*_ = *lb_j_* + *rand*(*ub_j_* − *lb_j_*) (2)

where *rand* is a random number uniformly distributed within the interval [0, 1], *lb_j_* and *ub_j_* denote the lower and upper bounds of the *j*-th dimension, respectively. After initialization, the fitness values for all individuals are calculated, and the individual with the highest fitness is selected as the initial dominant hippopotamus, or the population’s leader.

#### 3.1.2. Exploration Stage

This stage simulates the social structure and interactions of hippopotamus groups in water, corresponding to the algorithm’s global exploration behavior. A hippopotamus group consists of dominant males, females, and calves. Dominant males are responsible for protecting the territory and the group, while young males are expelled upon reaching adulthood and must establish their status through competition.

1. Male hippopotamus position update. The expelled young male hippopotamus attempts to challenge or attract females, and its position is influenced by the dominant hippopotamus’s location, as shown in Equation (3).
  (3) $$x_{i,j}^{\mathrm{Male}}=x_{i,j}+\mu_{1}\cdot \left({\mathrm{Leader}}_{j}-I_{1}\cdot x_{i,j}\right)$$
  where $x_{i,j}^{\mathrm{Male}}$ is the new position of the *i*-th male hippopotamus in the *j*-th dimension, *Leader_j_* is the position of the dominant hippopotamus in the *j*-th dimension, *μ*_1_ and *I*_1_ are random parameters used to control the step size and direction of movement, enhancing the randomness of the search.
2. Female and calf position update. Females and calves typically remain near the dominant male. However, the curiosity may cause calves to wander away from their mother or even leave the group. This behavior is simulated through a threshold *T* (generated by an exponential decay function) and a random number *r*_6_, as described by Equations (4) and (5).
  (4) $$x_{i,j}^{\mathrm{Female}}=\left\{\begin{array}{c} x_{i,j}+A_{1}\cdot ({\mathrm{Leader}}_{j}-I_{2}\cdot {\mathrm{MG}}_{i,j}),\;T>0.6\\ \Xi ,\;\mathrm{else}\; \end{array}\right.$$
  (5) $$\Xi =\left\{\begin{array}{c} x_{i,j}+A_{2}\cdot ({{\mathrm{MG}}_{i,j}-\mathrm{Leader}}_{j}),\;r_{6}>0.5\\ {\mathrm{lb}}_{j}+r_{7}\cdot ({\mathrm{ub}}_{j}-{\mathrm{lb}}_{j}),\;\mathrm{else}\; \end{array}\right.$$
  Here, *MG_i_*_,*j*_ denotes the average position of a randomly selected hippopotamus, *A*_1_ and *A*_2_ are randomly chosen coefficient vectors. When *T* > 0.6, it indicates that a calf moves away from its mother. When *T* ≤ 0.6 and *r*_6_ > 0.5, although the calf is away from its mother, it remains near the group. Otherwise, it has already detached from the group. This design enables the algorithm to perform local search around the leader while also allowing exploration of more distant regions.

#### 3.1.3. Exploitation Stage

This stage simulates the behavior of hippopotamuses fleeing to the nearest safety zone when unable to repel a predator, reflecting the algorithm’s local exploitation capability. A hippopotamus seeks a safer spot close to its current location, essentially performing a detailed local search.

1. Generate local search range. A contracting local search space is defined around the current hippopotamus position, as shown in Equation (6).
  (6) $${\mathrm{lb}}_{j}^{\mathrm{local}}=\frac{lb_{j}}{t},{\mathrm{ub}}_{j}^{\mathrm{local}}=\frac{{\mathrm{ub}}_{j}}{t},t=1,2,\ldots ,T$$
  where *t* is the current iteration number and *T* is the maximum number of iterations. As the iterations proceed, the search range gradually narrows, promoting convergence.
2. Position update. The hippopotamus attempts a new position within this local range, and the position update formula is given by Equation (7).
  (7) $$x_{i,j}^{\mathrm{HippoE}}=x_{i,j}+r_{10}\cdot ({\mathrm{lb}}_{j}^{\mathrm{local}}+s_{1}\cdot ({\mathrm{ub}}_{j}^{\mathrm{local}}-{\mathrm{lb}}_{j}^{\mathrm{local}}))$$
  where *r*_10_ is a random number in [0, 1], and *s*_1_ is a coefficient randomly selected from a predefined strategy set, used to control the direction and pattern of the local search. If the fitness of the new position $x_{i,j}^{\mathrm{HippoE}}$ is better than that of the original position, the hippopotamus’s position is updated; otherwise, it remains unchanged. This algorithm ensures deep exploitation within the potentially optimal regions.

#### 3.1.4. Local Optima Escape Stage

This stage simulates how a group of hippopotamuses reacts defensively when encountering predators such as lions or crocodiles, mirroring the algorithm’s ability to escape local optima and to enhance global exploration. When a predator approaches, a hippopotamus turns to face the threat and opens its massive jaws to intimidate.

1. Predator position modeling. A predator position is randomly generated within the search space to simulate an external threat, as shown in Equation (8).
  (8) $${\mathrm{Predator}}_{j}={\mathrm{lb}}_{j}+\vec{\tau_{s}}\cdot \left(ub_{j}-lb_{j}\right)$$
  where $\vec{\tau_{s}}$ is a random vector within the range [0, 1].
2. Distance-based defense response. The distance between the hippopotamus and the predator is calculated as *D* = |*Predator_j_* − *x_i_*_,*j*_|. Based on the distance and fitness, the hippopotamus adopts different defensive movement strategies, as formulated in Equation (9).
  (9) $$x_{i,j}^{\mathrm{HippoR}}=\left\{\begin{array}{c} \vec{\mathrm{RL}}⊕{\mathrm{Predator}}_{j}+\left(\frac{N}{\left(L-\mathrm{Acos}\left(2\pi \beta \right)\right)}\right)\cdot \left(\frac{1}{D}\right),\;F_{\mathrm{Predator}}<F_{i}\;\\ \vec{\mathrm{RL}}⊕{\mathrm{Predator}}_{j}+\left(\frac{N}{\left(L-\mathrm{Acos}\left(2\pi \beta \right)\right)}\right)\cdot \left(\frac{1}{2D+\tau_{s}}\right),\;\mathrm{else}\; \end{array}\right\}$$
  where $\vec{\mathrm{RL}}$ is a random flight vector obeying the Lévy distribution, capable of generating long step jumps to help individuals escape their current positions. *F_Predator_* and *F_i_* are the fitness values of the predator position and the current hippopotamus position, respectively. This mechanism encourages hippopotamuses to move substantially toward or away from the predator, effectively preventing the population from becoming trapped in local optima. If the fitness after movement decreases, the hippopotamus is considered captured and replaced by a new individual, thereby maintaining population diversity.

### 3.2. Logistic-Tent Hybrid Chaotic Mapping

In metaheuristic optimization algorithms, the initial population distribution significantly affects the algorithm’s global search capability and convergence speed. Traditional random initialization methods may lead to uneven population distribution, thereby reducing the algorithm’s exploration efficiency in complex solution spaces. Chaotic mappings are widely employed to generate initial populations with greater diversity due to their inherent ergodicity, aperiodicity, and sensitivity to initial conditions.

Among numerous chaotic mappings, the Logistic and Tent mappings represent two complementary chaotic systems. Logistic mapping is known for its simple structure and rich chaotic dynamics, which can generate highly unpredictable sequences that enhance the randomness and globality of the search. Tent mapping excels at achieving a uniform distribution under ideal conditions, enabling the generated sequences to cover the value range more evenly.

Logistic mapping is one of the most classic chaotic systems and is mathematically expressed by Equation (10).

*x_n_*_+1_ = *μx_n_*(1 − *x_n_*), *x_n_* ∈ (0, 1), *μ* ∈ [0, 4] (10)

When *μ* = 4, the system is in a fully chaotic state, and the sequence exhibits ergodicity within the (0,1) interval.

The expression of Tent mapping is shown in Equation (11).

(11) $$x_{n+1}=\left\{\begin{array}{c} \alpha x_{n}\;,x_{n}<0.5\;\\ \alpha \left(1-x_{n}\right),\;x_{n}\geq 0.5\; \end{array},\;x_{n}\in \left(0,1\right),\;\alpha \in (0,2]\right.$$

When *α* = 2, the system exhibits uniform chaotic behavior and is more ergodic than Logistic mapping.

However, single chaotic mappings have limitations. Logistic mapping is more likely to produce values near the boundaries, resulting in an uneven distribution. Tent mapping is uniformly distributed for parameter *α* = 2 but exhibits periodic windows and unstable periodic points. To overcome these shortcomings, this paper proposes a Logistic-Tent hybrid chaotic mapping that combines the advantages of both techniques to generate initial populations with a more uniform distribution and stronger randomness.

### 3.3. Trend-Aware Mechanism (TAM)

Traditional metaheuristic algorithms update individual positions based on the population state and random operators of the current iteration. Although this search approach provides some exploratory capability, it fails to exploit the search trajectory information embedded in historical iterations, resulting in a degree of blindness to the search direction. Consequently, in complex fitness landscapes, the algorithm may converge slowly or become trapped in local optima. To enhance the algorithm’s capacity to dynamically recognize and leverage search trends, this study introduces a Trend-Aware Mechanism (TAM), proposed by Lian et al. in 2025 [65]. It systematically records and analyzes historical position and fitness information for the population, quantifies the underlying trend in the search process, and intelligently guides individual updates. As a result, TAM significantly improves the algorithm’s search efficiency and convergence accuracy.

The implementation of TAM relies on two historical information matrices. The first matrix is the historical search position matrix, denoted *search*_*history*, with dimensions *N* × *Max*_*iter* × *dim*, where *N* is the number of individuals in the population, *Max*_*iter* is the maximum number of iterations, and *dim* is the dimensionality of the optimization problem. This three-dimensional matrix stores the positions of all individuals in the population at each iteration. The second matrix is the historical fitness matrix, denoted as *fitness*_*history*, with dimensions *N* × *Max*_*iter*. This two-dimensional matrix records the fitness values corresponding to the search positions. To ensure the reliability of trend analysis, the mechanism imposes an iteration constraint. It activates only when *i* > *n*, ensuring that the algorithm has at least *n* generations of historical data, avoiding computational instability or misjudging trends caused by insufficient historical information.

### 3.4. Improved Hippopotamus Optimization Algorithm (IHOA)

This paper integrates the two strategies into the original HOA search framework and proposes the Improved Hippopotamus Optimization Algorithm (IHOA). On the one hand, the Logistic-Tent hybrid chaotic mapping enhances the ergodicity and uniformity of the initial population distribution. This improvement allows the algorithm to start the search from a diverse set of higher-quality initial points, effectively alleviating early exploration blind spots caused by random initialization. On the other hand, the trend-aware mechanism (TAM) provides directional guidance and adaptive corrections across the algorithm’s exploration, exploitation, and local optima escape stages. It significantly enhances the intelligence and convergence efficiency of the search process by recording and analyzing historical search trajectories. The synergistic effect of the two strategies provides IHOA with stronger global optimization capability, faster convergence, and a higher escape rate from local optima, while maintaining the simplicity of the original HOA framework, thereby laying a solid algorithmic foundation for subsequent hyperparameter optimization of the CNN-BiLSTM model.

#### 3.4.1. Population Initialization with Hybrid Chaotic Mapping

The standard HOA adopts a random strategy to generate the initial population, leading to an uneven distribution of individuals within the solution space and resulting in local clusters and void regions. This strategy reduces the population’s traversal and coverage uniformity across the search space, thereby impairing the exploration efficiency and global optimization capability in the early stages of the algorithm. Therefore, this paper introduces the Logistic-Tent hybrid chaotic mapping to address this limitation of random initialization. This hybrid mapping combines Logistic mapping and Tent mapping through a dynamic weight. Let *x_n_* be the current chaotic sequence value. The mathematical definition of the Logistic-Tent hybrid chaotic mapping is given by Equation (12).

(12) $$x_{n+1}=\left\{\begin{array}{c} \left[\lambda \cdot \mu x_{n}(1-x_{n})+\left(1-\lambda \right)\cdot 2x_{n}\right]\;\mathrm{mod}1,\;x_{n}<0.5\\ \left[\lambda \cdot \mu x_{n}\left(1-x_{n}\right)+\left(1-\lambda \right)\cdot 2\left(1-x_{n}\right)\right]\;\mathrm{mod}1,\;x_{n}\geq 0.5 \end{array}\right.$$

(13) $$\lambda =0.5+0.3\cdot \sin\left(\frac{2\mathrm{\pi n}}{N}\right)$$

Here, *μ* = 4 ensures that the Logistic branch is in a fully chaotic state, and *λ* is the adaptive mixing weight, dynamically adjusted according to the iteration progress, as shown in Equation (13). This design facilitates hybrid mapping to highlight various chaotic characteristics at different stages of the iteration process. In the early stage, the mixing weight is dynamically adjusted based on the iteration progress, allowing the hybrid mapping to balance the characteristics of the Logistic and Tent mappings.

**Figure 2:**
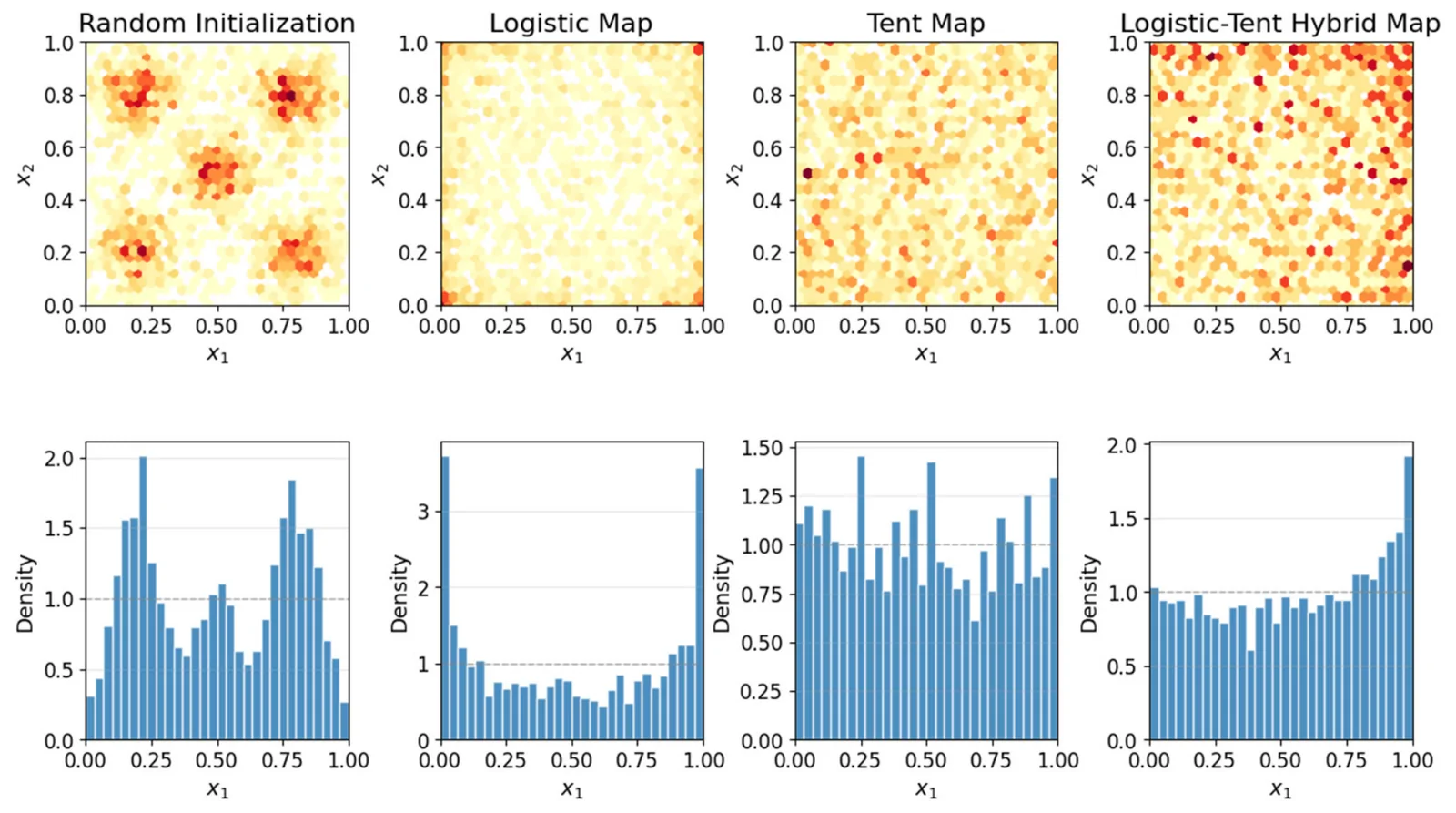
The particle distribution diagrams of the four initialization methods.

To visually demonstrate the superiority of the hybrid chaotic mapping, four initialization strategies are compared using two-dimensional density distributions and one-dimensional marginal histograms. As shown in Figure 2, the random initialization exhibits clear clusters and voids. The logistic mapping is highly concentrated at the boundaries, while the center remains sparse. The tent mapping displays regular stripes and spikes due to periodic windows. In contrast, the Logistic-Tent hybrid chaotic mapping yields a completely uniform distribution, with no aggregation or voids. This comparison demonstrates that the hybrid strategy successfully addresses the drawbacks of single-mapping strategies and produces a high-quality initial population.

During the population initialization stage, IHOA substitutes the standard HOA’s random initialization with the Logistic-Tent hybrid chaotic mapping. The initial position of the *j*-th dimension for the *i*-th individual is generated according to Equations (12) and (13) and can be uniformly expressed as Equation (14).

(14) $$x_{i,j}=l{b_{j}+\xi }_{i,j}\cdot \left(ub_{j}-lb_{j}\right)$$

where *ξ_i_*_,*j*_ ∈ (0,1) is the chaotic sequence value generated by the hybrid chaotic mapping, and *lb_j_* and *ub_j_* are the lower and upper bounds of the *j*-th dimension variable, respectively. Compared with random numbers following a uniform distribution, the sequences generated by the hybrid chaotic mapping exhibit superior ergodicity and uniformity of distribution. The Logistic-Tent hybrid chaotic mapping enables the initial population to span a wider range and achieve a more balanced distribution within the solution space. As a result, it provides a higher-quality set of starting points for the subsequent iterative search. After initialization is complete, the fitness values for all individuals are calculated, and the initial dominant individual, known as the Leader, is identified. Meanwhile, the historical search position matrix and the historical fitness matrix are initialized to establish a solid data foundation for the upcoming use of the trend-aware mechanism.

#### 3.4.2. Trend-Aware Mechanism Improves HOA

The position updates of the standard HOA in the three stages primarily rely on the current population state and random operators. This ineffective use of historical search trajectories introduces a degree of blindness in the search direction. Consequently, in complex fitness landscapes, the algorithm is prone to problems such as declining population diversity in later iterations, slower convergence, and local optima trapping. To overcome these limitations, this paper integrates the trend-aware mechanism (TAM) as an intelligent guidance operator across all three position-update stages of HOA. At the operational level, TAM does not replace HOA’s original position update formulas. Instead, it functions as a post-processing correction applied to the candidate positions generated at each stage. Specifically, in each iteration, the HOA generates one or more candidate positions for every individual in the population based on the current stage. If the activation condition related to the iteration count is met, the TAM is invoked to correct the candidate positions. Otherwise, the TAM correction step is skipped, and the positions created by the HOA are used directly for fitness evaluation.

The specific implementation steps of the TAM are described as follows.

1. Selection of Historical Trend Points.

After accumulating sufficient historical data, TAM constructs linear trajectories of individual historical movements and selects historical search points highly correlated with the evolutionary trend. The positions of individual *j* at iteration *i* − 1, *search*_*history*(*j*,*i* − 1,:), and at iteration *i* − 2, *search*_*history*(*j*,*i* − 2,:), are selected to construct a trend line. To reduce computational complexity, only the latest *n* historical search data points are considered. The distance *d* from each historical point *P* to this trend line is calculated, and the *K* historical points with the smallest distances are selected as the trend analysis samples. The calculation formula for the distance *d* from a historical search point *P* to the line is given by Equation (15).

(15) $$d(P)=\frac{\left\|(\mathrm{search}\_\mathrm{history}(j,i-1,:)-P)\times (\mathrm{search}\_\mathrm{history}(j,i-2,:)-\mathrm{search}\_\mathrm{history}(j,i-1,:))\right\|}{\left\|\mathrm{search}\_\mathrm{history}(j,i-2,:)-\mathrm{search}\_\mathrm{history}(j,i-1,:)\right\|}$$

Distance-based filtering effectively preserves historical information that aligns with the population’s evolutionary trend. At the same time, redundant search data are eliminated, thereby improving trend-judgment accuracy and computational efficiency.

2. Generation of Adaptive Covariance Vector.

In high-dimensional search spaces, if the offset vector is generated solely based on historical trends, the search process may lose diversity due to excessive reliance on existing directions, leading to premature convergence or entrapment in local optima. To address this issue, TAM introduces an adaptive covariance mechanism that leverages population distribution characteristics to generate a high-dimensional trend vector that balances randomness and stability.

First, the basic trend vector *V* pointing toward historical positions is calculated as shown in Equation (16).

*V* = *search*_*history*(*j*,*i* − 2,:) − *search*_*history*(*j*,*i* − 1,:) (16)

Subsequently, the covariance matrix *Σ* is computed based on the current population position distribution to capture the overall spatial distribution characteristics of the population, as defined in Equation (17).

(17) $$\Sigma =\frac{1}{N}\sum_{j=1}^{N}((X\left(j,:\right)-\mu )(X\left(j,:\right)-{\mu )}^{T})$$

*X*(*j*,:) = *search*_*history*(*j*,*i* − 1,:) (18)

Here, *μ* is the mean vector of the current population positions. Using the basic trend vector *V* as guidance and incorporating a random vector *Z* sampled from a multivariate Gaussian distribution with zero mean and covariance matrix *Σ*, the adaptive offset vector *V*′ is constructed as shown in Equation (19).

*V*′ = *αV* + *βZ* (19)

*α* and *β* in the equation are dynamic weighting coefficients that balance the guidance of historical trends and the capability for random exploration. Their calculation formulas are given by Equation (20).

(20) $$\alpha =\frac{i}{\text{Max\_iter}},\;\beta =1-\alpha$$

To ensure that the step size of the offset vector remains consistent with the historical trend, *V*′ is normalized as shown in Equation (21).

(21) $$V^{\prime }=\frac{V^{\prime }}{\left\|V^{\prime }\right\|}\cdot \left\|V\right\|$$

This step enables the algorithm to emphasize random global exploration in early iterations and strengthen historical trend guidance in later iterations, thereby achieving an adaptive dynamic adjustment of the optimization strategy.

3. Fitness-Driven Position Update.

To determine the final search update direction, a fitness comparison analysis is conducted on the selected *K* neighboring historical points. Let *f_i_* represent the fitness value of the *i*-th historical trend point. This value is compared with the fitness values of individual *j* from the previous two iterations, specifically *fitness*_*history*(*j*,*i* − 1,:) and *fitness*_*history*(*j*,*i* − 2,:). This comparison is used to create a fitness judgment indicator *F_i_*, as defined in the following cases.

- If f*_i_* > max(*fitness*_*history*(*j*,*i* − 1,:), *fitness*_*history*(*j*,*i* − 2,:)), then set *F_i_* = 1.
- Else if *f_i_* > min(*fitness*_*history*(*j*,*i* − 1,:), *fitness*_*history*(*j*,*i* − 2,:)) and *fitness*_*history*(*j*,*i* − 1,:) < *fitness*_*history*(*j*,*i* − 2,:), then set *F_i_* = 0.4.
- Else if *f_i_* > min(*fitness*_*history*(*j*,*i* − 1,:), *fitness*_*history*(*j*,*i* − 2,:)) and *f_i_* < max(*fitness*_*history*(*j*,*i* − 1,:), *fitness*_*history*(*j*,*i* − 2,:)), then set *F_i_* = 0.
- Else if *f_i_* < min(*fitness*_*history*(*j*,*i* − 1,:), *fitness*_*history*(*j*,*i* − 2,:)), then set *F_i_* = −1.

The sum of the judgment indicators for all trend points, *ΣF_i_*, is calculated, and a differentiated position update strategy is executed based on the result. When *ΣF_i_* > 0, it indicates that there exists a poor fitness valley between the two points, implying that the current search direction is likely to trap the algorithm in a degraded region; thus, a reverse adjustment is applied to evade this local pitfall. The update vector is computed as shown in Equations (22) and (23).

(22) $$\mathrm{Vec}=V^{\prime }\cdot \frac{1-i/\text{Max\_iter}}{10}\;$$

*X_new_*(*j*,:) = *X_new_*(*j*,:) − *Vec* (23)

When *ΣF_i_* < 0, it indicates that the higher fitness values are likely located between the two historical points, suggesting that moving along the historical trend direction may lead to better regions. The update vector is computed as shown in Equation (24).

*X_new_*(*j*,:) = *X_new_*(*j*,:) + *Vec* (24)

Through the three steps above, the TAM achieves trend-aware correction of candidate positions. In the global exploration stage, male, female, and calf hippopotamuses generate candidate positions according to Equations (3)–(5). If the activation condition *t* > *n* is met, TAM applies trend-guided correction to the candidate solutions based on historical search trajectories, enabling the exploration process to maintain randomness while remaining aware of convergence toward favorable regions. During the local exploitation stage, hippopotamus individuals establish tentative positions within the contracting local space using Equation (7). The TAM intervention performs trend-aware fine-tuning on candidate solutions, thereby enhancing the algorithm’s capability to refine excavation in potentially optimal regions. In the local optima escape stage, as described by Equations (8) and (9), TAM uses trend analysis and fitness comparisons to help individuals make more informed decisions about escape direction, thereby improving the population’s success rate in leaving low-quality regions. The full-coverage design across all three stages ensures that TAM optimizes the algorithm’s convergence behavior holistically, rather than being restricted to local improvements within a single search phase.

The values of the TAM activation threshold *n* and the number of neighboring points *K* are selected to balance trend responsiveness, local information reliability, and computational efficiency. Specifically, the setting *n* = 5 allows TAM to accumulate sufficient historical search data before activation, thereby reducing the impact of short-term fluctuations and enabling timely adaptation to trends. Meanwhile, *K* = 3 provides a compact set of neighboring points for estimating local search tendencies without introducing excessive computational overhead or over-smoothing the local trend. These settings offer a practical balance between the effectiveness and efficiency of TAM and are therefore adopted in this study.

In conclusion, the conditional embedding strategy ensures that the algorithm performs unbiased global exploration in the early iterations. In addition, it leverages trend-perception capabilities to accelerate convergence and avoid local traps once sufficient historical information has been accumulated.

#### 3.4.3. The Steps of IHOA

The pseudocode in Algorithm 1 presents the IHOA’s overall logic and functionality.
**Algorithm 1** IHOA (Improved Hippopotamus Optimization Algorithm)
**Input**: Population size N, Maximum iterations T, Dimensionality D,       Lower bound vector lb, Upper bound vector ub,      TAM activation threshold n, Number of nearest points K**Output**: Best solution X_best and its fitness f_best 1:   Initialize population X = {X_1_, X_2_, ..., X_N} using Logistic-Tent hybrid chaotic map 2:   Evaluate fitness f(X_i_) for each X_i_ ∈ X 3:   Identify initial leader Leader with best fitness f_best 4:   Initialize search_history and fitness_history matrices 5:   for t = 1 to T do 6:    Update dynamic parameters (e.g., contraction factor t) 7:    for each individual X_i_ ∈ X do 8:        // Phase 1: Exploration stage 9:        Generate candidate position X_cand using Equations (3)–(5)10:       if t > n then11:           Apply TAM correction to X_cand to obtain X_tam12:           Select a better position between X_cand and X_tam as X_new13:       else14:           X_new ← X_cand15:       end if16:       Evaluate f(X_new); update X_i_ if f(X_new) is better17:       // Phase 2: Exploitation stage18:       Generate local candidate X_local using Equation (7)19:       if t > n then20:           Apply TAM correction to X_local to obtain X_tam_local21:           Select a better position between X_local and X_tam_local as X_new_local22:       else23:           X_new_local ← X_local24:       end if25:       Evaluate f(X_new_local); update X_i_ if f(X_new_local) is better26:       // Phase 3: Local optima escape stage27:       Generate defensive position X_def using Equations (8) and (9)28:       if t > n then29:           Apply TAM correction to X_def to obtain X_tam_def30:           Select a better position between X_def and X_tam_def as X_new_def31:       else32:           X_new_def ← X_def33:       end if34:       Evaluate f(X_new_def)35:       if f(X_new_def) is better than f(X_i_) then36:           Update X_i_ ← X_new_def37:       else38:           Regenerate X_i_ randomly if captured by a predator39:       end if40:   end for41:   Update search_history and fitness_history with current X and f(X)42:   Update global best solution X_best and f_best43:    end for

As shown in Algorithm 1, the Logistic-Tent hybrid chaotic initialization is implemented solely during the algorithm’s startup phase (line 1), yielding a significant improvement in the quality of the initial population at minimal computational cost. TAM is invoked independently in three stages of HOA, confirming its comprehensive coverage of all search phases. As a plug-and-play intelligent module, TAM is selectively activated at every stage of each iteration once candidate positions have been generated. Its primary function is to refine a tentative position in accordance with the HOA’s inherent rules. TAM analyzes the historical search trajectories of that individual and its neighbors, applying direction-aware fine-tuning to enhance the tentative position. Without compromising the HOA’s exploration–exploitation balance, this fine-tuning effectively addresses its deficiency in search-direction awareness. It enables the algorithm to navigate around local optima more smoothly and accelerate in favorable gradient directions within complex fitness landscapes.

In summary, by integrating hybrid chaotic initialization with a pervasive trend-aware mechanism, IHOA achieves a threefold improvement over the standard HOA in the quality of search starting points, the directionality of the search process, and the dynamic balance between exploration and exploitation.

### 3.5. IHOA-CNN-BiLSTM Model

To effectively identify local anomalous features and global semantic inconsistencies in fake information texts, this paper constructs a parallel dual-channel hybrid deep learning model that combines Convolutional Neural Networks (CNNs) and Bidirectional Long Short-Term Memory (BiLSTM) networks, referred to as CNN-BiLSTM. The model consists of an input layer, an embedding layer, a parallel CNN feature-extraction channel, a BiLSTM sequence-modeling channel, a feature-fusion layer, and an output layer.

The input text is preprocessed and represented as a fixed-length word index sequence, which is then mapped into a word embedding matrix through the embedding layer. The word embedding matrix is simultaneously fed into the two parallel feature-extraction channels. In the CNN channel, multiple one-dimensional convolutional kernels of varying sizes are employed to extract local n-gram features. After max pooling, a local semantic representation vector is obtained. In the BiLSTM channel, forward and backward LSTM networks encode long-range dependencies of the sequence from both directions. The final hidden states from the two directions are concatenated to create a global contextual representation vector. Subsequently, the output vectors from the two channels are connected to form a fused feature vector, which is passed through a fully connected layer. Finally, a Sigmoid function is used to output the probability of the binary classification. This parallel dual-channel design enables the model to simultaneously characterize textual features from local semantic patterns and global contextual dependencies. Additionally, the two branches collaborate to enhance the model’s discriminative ability against fake information.

Since the performance of the CNN-BiLSTM model greatly depends on appropriate hyperparameter configurations, traditional manual tuning cannot guarantee global optimality. Consequently, this paper employs the IHOA to automatically optimize these hyperparameters. In IHOA, the above hyperparameters are encoded as the position vector of a hippopotamus individual. Each dimension corresponds to a variable to be optimized, with suitable mappings applied based on the variable’s hyperparameter type. For discrete hyperparameters, the continuous search position is normalized and divided into equal intervals corresponding to the candidate values. The candidate value associated with the interval containing the position is selected. For continuous hyperparameters, the position is directly mapped to the predefined hyperparameter range. The classification accuracy of the CNN-BiLSTM model on the validation set is utilized as the fitness function. During the iterative process, IHOA continuously generates and evaluates different hyperparameter combinations. Leveraging the high-quality initial population generated by the Logistic-Tent chaotic initialization with the intelligent guidance of the trend-aware mechanism, the algorithm efficiently converges to optimal hyperparameter configurations.

### 3.6. Time Complexity Analysis

To analyze the computational cost of the proposed IHOA-CNN-BiLSTM framework, the time complexity of the IHOA optimization process and the CNN-BiLSTM fitness evaluation process is analyzed using Big-O notation. Let *N* denote the population size, *T* the maximum number of iterations, and *D* the dimensionality of the search space.

In IHOA, the Logistic-Tent hybrid chaotic mapping generates the initial population in time complexity *O*(*ND*). During each iteration, the HOA updates the positions of *N* individuals, resulting in a basic search complexity of *O*(*ND*). TAM also conducts historical trend analysis and calculates the covariance matrix, with a primary computational complexity of *O*(*ND*^2^). Therefore, the overall time complexity of IHOA over *T* iterations can be expressed as *O*(*TND*^2^). The *D*^2^ term mainly results from the covariance matrix calculation in TAM. Since the search dimensionality *D* is relatively small in this study, the additional computational cost introduced by TAM remains manageable.

In the IHOA-CNN-BiLSTM framework, each individual in IHOA represents a set of CNN-BiLSTM hyperparameters, and its fitness is evaluated using validation accuracy. Let *L* denote the input sequence length, *C* the number of CNN kernels, *K* the convolution kernel size, *H* the number of BiLSTM hidden units, *M* the number of training samples, and *R* the number of training epochs. The main computational complexity of training a CNN-BiLSTM model can be estimated as Equation (25).

(25) $$O\left[R\frac{M}{B}\left(\mathrm{LCKE}+\mathrm{LH}\left(E+H\right)\right)\right]$$

Here, *B* denotes the batch size. Therefore, considering the evaluation of *N* candidate solutions over *T* iterations, the overall time complexity of the proposed IHOA-CNN-BiLSTM framework can be expressed as Equation (26).

(26) $$O\left[\mathrm{NTR}\frac{M}{B}\left(\mathrm{LCKE}+\mathrm{LH}\left(E+H\right)\right)+\mathrm{TN}D^{2}\right]$$

The first term primarily reflects the repeated fitness evaluation of the CNN-BiLSTM model, while the second term represents the computational cost of the IHOA optimization process. Therefore, the main computational cost of the proposed framework arises from training models for candidate hyperparameter configurations, whereas the additional optimization cost introduced by IHOA remains relatively small.

## 4. Experiments

### 4.1. Experimental Setup

#### 4.1.1. Experimental Environment

This study conducts all experiments on a server equipped with two NVIDIA RTX 4080S GPUs in MATLAB 2024b. The CNN-BiLSTM hybrid model is constructed in MATLAB using the Deep Network Designer, and hyperparameter optimization is accelerated with parallel computing resources. The hyperparameter search space used by IHOA is predefined, with validation accuracy serving as the fitness criterion during optimization. The specific search ranges and other experimental settings are summarized in Table 1. Because IHOA is a stochastic population-based optimization algorithm, the selected hyperparameter configurations may vary across independent runs. Therefore, rather than reporting a single fixed configuration from a single run, this study presents the complete hyperparameter search space and optimization settings. Together with the publicly available source code, these details facilitate reproduction of the hyperparameter optimization procedure under the same experimental conditions.

#### 4.1.2. Evaluation Indicators

This study uses accuracy, precision, recall, and F1-score as evaluation metrics to assess the model. Accuracy refers to the proportion of correctly predicted samples among all samples. Precision is defined as the proportion of actual positive samples among those predicted as positive. Recall measures the proportion of actual positive samples correctly predicted as positive. F1-score is the harmonic mean of precision and recall, providing a combined measure of classification performance.

#### 4.1.3. Data Sources and Preprocessing

This study uses two publicly available datasets of fake information, namely the PolitiFact subset of FakeNewsNet [66] and the Weibo21 [67] dataset. FakeNewsNet is a widely used benchmark dataset for fake news detection, and its PolitiFact subset contains political news labeled as fake or real. The dataset used in this study comprises 432 fake news items and 624 real news items, for a total of 1056 samples.

The Weibo21 dataset, constructed by Nan et al. in 2021 [67], is designed for cross-domain research on fake information detection. It contains 4488 fake and 4640 real information items across nine distinct domains. The dataset features clear domain labels and a balanced category distribution, making it suitable for evaluating models’ generalization across different topics.

During preprocessing, duplicate and invalid records are removed, and samples with missing textual content are excluded. The remaining samples are labeled as either fake or real and subsequently divided into training, validation, and test sets using a stratified sampling strategy in a 7:1:2 ratio. The three subsets are mutually exclusive to prevent data leakage. The training set is used for model training, the validation set is used for hyperparameter optimization, and the test set is reserved exclusively for final performance evaluation. For the PolitiFact data, we use 300d GloVe embeddings with word-level tokenization. For the Weibo21 data, we apply Jieba word segmentation and use 300d Word2Vec embeddings. For both datasets, all sequences are either padded or truncated to a fixed length of 256.

### 4.2. Comparative Experiments

#### 4.2.1. Baseline Models Comparative Experiments

To verify the effectiveness of the proposed IHOA in optimizing the hyperparameters of the CNN-BiLSTM model, comparative experiments are conducted on both the PolitiFact and the Weibo21 datasets. The compared models include the CNN-BiLSTM, Random Search-CNN-BiLSTM, Bayesian Optimization-CNN-BiLSTM, TPE-CNN-BiLSTM, Hyperband-CNN-BiLSTM, GWO-CNN-BiLSTM, WOA-CNN-BiLSTM, BWO-CNN-BiLSTM (BWO: Beluga Whale Optimization), BKA-CNN-BiLSTM, HOA-CNN-BiLSTM, BERT, RoBERTa, and the proposed IHOA-CNN-BiLSTM. To improve the reliability of the comparative results, each method is independently executed 30 times under the same search space, data partition, fitness function, and training settings. Table 2 presents the mean ± standard deviation of the four evaluation metrics over the 30 runs. In addition, the Wilcoxon signed-rank test is performed on the 30 Accuracy values for the IHOA-CNN-BiLSTM against each of the other methods, with Holm correction for multiple comparisons. The experimental results are detailed in Table 2.

On the PolitiFact dataset, the three categories of models exhibit a clear performance hierarchy. Firstly, all conventional hyperparameter optimization methods outperform the unoptimized CNN-BiLSTM, indicating that systematic hyperparameter search effectively improves the CNN-BiLSTM architecture’s performance. In particular, the Hyperband-CNN-BiLSTM model exceeds conventional baselines due to its effective resource allocation mechanism. Secondly, the metaheuristic-based methods also surpass the unoptimized model. However, considerable variation exists among different algorithms, suggesting that relying solely on a population-based search mechanism does not ensure the discovery of the optimal hyperparameter combination. Thirdly, among all CNN-BiLSTM hybrid models, IHOA-CNN-BiLSTM achieves the highest performance, with 90.13% accuracy and 88.02% F1-score, which are 2.36 and 2.76 points higher than those of HOA-CNN-BiLSTM. The results confirm that the Logistic-Tent hybrid chaotic initialization improves the coverage quality of the initial population in the search space. At the same time, the trend-aware mechanism further exploits historical search trajectories to provide directional corrections for candidate solutions. TAM enables our model to continue discovering superior configurations even beyond the already strong baselines of Hyperband and HOA. Finally, regarding pretrained language models, IHOA-CNN-BiLSTM outperforms BERT by 1.88 percentage points in accuracy and 2.24 points in F1-score. RoBERTa achieves 91.02% accuracy and 89.11% F1-score, which are 0.89 and 1.09 percentage points higher than our model, respectively. However, RoBERTa-base has approximately 125 million parameters. In contrast, IHOA-CNN-BiLSTM contains only about 15 million trainable parameters. The Wilcoxon test indicates that the performance difference between the two is not statistically significant. IHOA-CNN-BiLSTM achieves competitive performance while remaining lightweight, making it suitable for resource-constrained scenarios despite the significant gap in model size and computational cost.

On the Weibo21 dataset, the performance discrepancies among the three baseline categories further reveal the different adaptability of optimization strategies to complex data distributions. Firstly, traditional hyperparameter optimization methods consistently outperform the unoptimized CNN-BiLSTM. In particular, Hyperband-CNN-BiLSTM exceeds GWO-CNN-BiLSTM, WOA-CNN-BiLSTM, BWO-CNN-BiLSTM, and BKA-CNN-BiLSTM. This result demonstrates that Hyperband’s resource-adaptive allocation is effective at identifying high-potential hyperparameter combinations. Secondly, metaheuristic-based models surpass the original CNN-BiLSTM, with HOA-CNN-BiLSTM achieving 83.18% accuracy. IHOA-CNN-BiLSTM further improves this to 88.18%, a gain of 5.00 percentage points over HOA. Thirdly, a comparison with pretrained language models shows that IHOA-CNN-BiLSTM surpasses BERT by 2.75 and 2.68 percentage points in accuracy and F1-score, respectively, and exceeds RoBERTa by 1.81 percentage points in both metrics. Notably, while RoBERTa achieves the highest performance on PolitiFact, IHOA-CNN-BiLSTM outperforms RoBERTa on Weibo21. This result indicates that superior performance is not solely due to larger parameter scales but is also closely related to the alignment of hyperparameter configurations with data distributions.

The performance differences between the two datasets arise from their distinct data characteristics. PolitiFact contains more structured political news with clearer textual and semantic patterns, whereas Weibo21 consists of shorter, informal, and heterogeneous social media content with greater linguistic noise. These differences make feature extraction and hyperparameter optimization more challenging on Weibo21, resulting in relatively lower performance. Overall, the results indicate that IHOA-CNN-BiLSTM is effective across both datasets, although its performance varies with the underlying data distribution and complexity.

#### 4.2.2. Computational Complexity Analysis

To evaluate the practical efficiency of IHOA, we compare the final CNN-BiLSTM models optimized by GWO, WOA, BWO, BKA, HOA, and IHOA in terms of Accuracy, F1-score, number of parameters (Params), and floating-point operations (FLOPs) on the Weibo21 dataset. All models are trained and tested under identical experimental settings. The results are reported in Table 3.

As the algorithm’s optimization capability improves, Params and FLOPs gradually increase, and detection performance generally improves. IHOA achieves the highest Accuracy and F1-score, with Params of 15.0 M and FLOPs of 30.1 G. Compared with HOA, IHOA increases Params from 14.2 M to 15.0 M and FLOPs from 28.5 G to 30.1 G, while Accuracy and F1-score improve from 83.18 to 88.18 and from 82.54 to 87.94, respectively. Compared with GWO, both the increases in Params and FLOPs are moderate, whereas the performance gains are substantial. Although TAM introduces an additional O(ND^2^) complexity due to the covariance matrix, it is activated only after sufficient historical information has been accumulated and only corrects candidate positions, so its impact on the final deployed model complexity is limited. Overall, IHOA achieves a favorable balance between computational cost and detection performance, supporting its practical efficiency in fake information detection.

### 4.3. Ablation Experiments

To systematically evaluate the independent contributions and synergistic effects of each improvement strategy in the IHOA on final detection performance, an ablation study is designed on the Weibo21 dataset. In this experiment, key components are gradually removed to analyze their impact on overall performance. The ablation study consists of three variant configurations.

w/o LT: removal of the Logistic-Tent hybrid chaotic initialization and restoration to standard random initialization, evaluating the impact of the hybrid chaotic initialization strategy on model performance.

w/o TAM: removal of the trend-aware mechanism, retaining only the hybrid chaotic initialization and the standard HOA search strategy, evaluating the impact of the trend-aware mechanism on model performance.

w/o TAM and LT: simultaneous removal of both the trend-aware mechanism and the hybrid chaotic initialization, evaluating the impact of removing both improvements on model performance.

The three variants above are compared with the complete IHOA-CNN-BiLSTM model under the same experimental environment, and each configuration is independently executed 30 times. Table 4 reports the mean ± standard deviation of the four evaluation metrics across the 30 runs, along with the *p*-values from the Wilcoxon signed-rank test comparing each variant to the full model based on the 30 Accuracy values. The results are presented in Table 4. The bold indicates the best performance.

The ablation study results indicate that both the Logistic-Tent hybrid chaotic initialization and the trend-aware mechanism significantly improve the model’s overall performance.

w/o TAM and LT: After removing both the trend-aware mechanism and the hybrid chaotic initialization, the accuracy, precision, recall, and F1-score decrease by 4.47, 4.31, 4.60, and 4.46 percentage points, respectively. Additionally, the Accuracy standard deviation is 0.86, which is noticeably larger than that of the full model. The model’s performance declines substantially, indicating that the two improvement strategies complement each other in IHOA. The hybrid chaotic initialization improves population quality by refining the search starting point, while the trend-aware mechanism enhances directionality and escape from local optima. The two strategies operate at different stages of optimization, and their collaborative interaction provides IHOA with stronger global optimization capability and higher convergence accuracy in high-dimensional hyperparameter spaces.

w/o TAM: After removing the trend-aware mechanism, the accuracy, precision, recall, and F1-score decrease by 3.73, 4.03, 3.56, and 3.80 percentage points, respectively. Its Accuracy standard deviation is 0.73, still higher than that of the full model. By systematically recording and analyzing the population’s historical search trajectories, the trend-aware mechanism provides pervasive directional guidance across the three stages of HOA. After removing this mechanism, the algorithm degenerates into a memoryless search that relies solely on the current iteration state. It loses the ability to perceive optimization trends in complex fitness landscapes and easily falls into local optima as population diversity declines in later iterations. This module has the greatest impact on precision, indicating that the hyperparameter combination derived from trend-aware guidance achieves a better balance between recall and F1-score.

w/o LT: Removing the hybrid chaotic initialization results in decreases of 2.99 percentage points in accuracy, 3.18 percentage points in precision, 2.66 percentage points in recall, and 2.92 percentage points in F1-score. Its Accuracy standard deviation is 0.67, the closest to the full model among the three variants. By combining the ergodicity advantages of Logistic and Tent mappings, the hybrid chaotic initialization strategy more evenly distributes the initial population across the solution space, providing higher-quality starting points for the subsequent search. After removing this strategy, the uneven distribution of the randomly initialized population may cause the algorithm to miss potentially high-quality regions early in the search, thereby degrading the quality of the final hyperparameter configuration.

The results of the ablation study demonstrate that the Logistic-Tent hybrid chaotic initialization and the trend-aware mechanism both positively contribute to IHOA’s performance. Both elements are essential and constitute the core technical advantages of the proposed algorithm.

### 4.4. Algorithm Convergence Analysis

**Figure 3:**
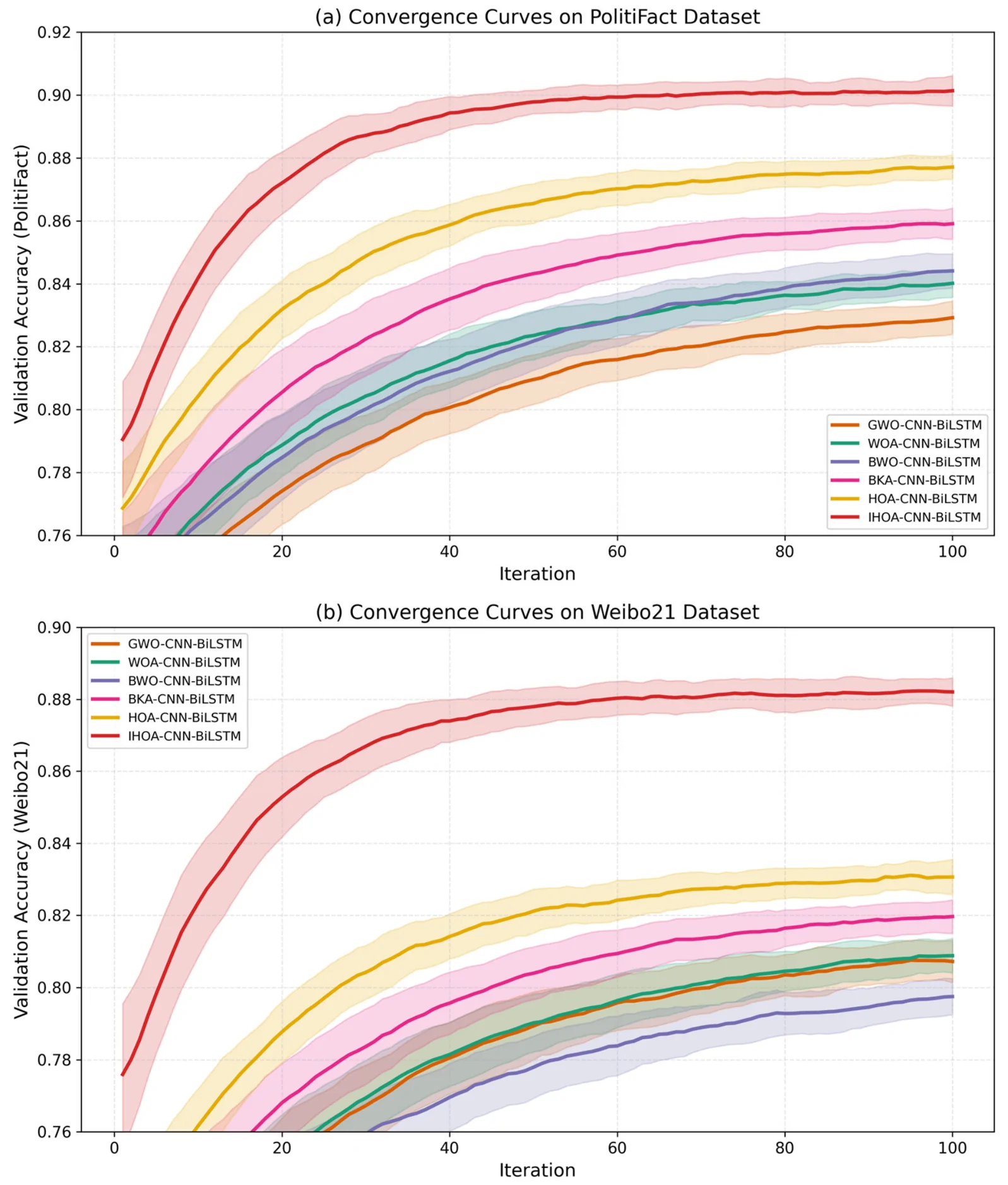
Convergence curve comparison results on the PolitiFact and Weibo21 datasets.

To intuitively evaluate the convergence performance of IHOA in the hyperparameter optimization task of CNN-BiLSTM, this experiment presents the fitness convergence curves of IHOA alongside five representative swarm intelligence optimization algorithms (HOA, GWO, WOA, BWO, and BKA) over 30 independent runs, where the solid lines represent the mean validation accuracy and the shaded areas denote the standard deviation. All algorithms are executed under identical experimental conditions, with a population size of *N* = 50 and a maximum number of iterations of *T* = 100. The fitness function is defined as the classification accuracy of the CNN-BiLSTM model on the validation set of the PolitiFact and Weibo21 datasets. The convergence curves are displayed in Figure 3.

As shown in Figure 3, across both datasets, the fitness starting point of IHOA in the early iterations is notably higher than that of the other compared algorithms. This advantage stems from the use of the Logistic-Tent hybrid chaotic initialization, which provides the population with a more uniform and ergodic initial distribution. As a result, the search begins in higher-quality regions. In the middle iterations, IHOA exhibits faster convergence and consistently maintains a superior fitness value. These results suggest that the trend-aware mechanism effectively guides positional updates throughout the three search stages. Additionally, it minimizes fitness fluctuations arising from random search by analyzing historical search trajectories. In the final iterations, IHOA achieves the highest accuracy, and its curve shows greater stability, as reflected in a consistently narrower standard deviation band than other methods. At the same time, the standard HOA and some of the other algorithms exhibit varying degrees of stagnation or slight oscillations in the later stages. This situation indicates that the adaptive covariance perturbation of IHOA effectively delays the decline in population diversity and prevents premature convergence. Overall, the convergence curve analysis verifies the superiority of the proposed improvement strategies in terms of efficiency and accuracy.

### 4.5. Hyperparameter Optimization Analysis

#### 4.5.1. Confusion Matrix Analysis

**Figure 4:**
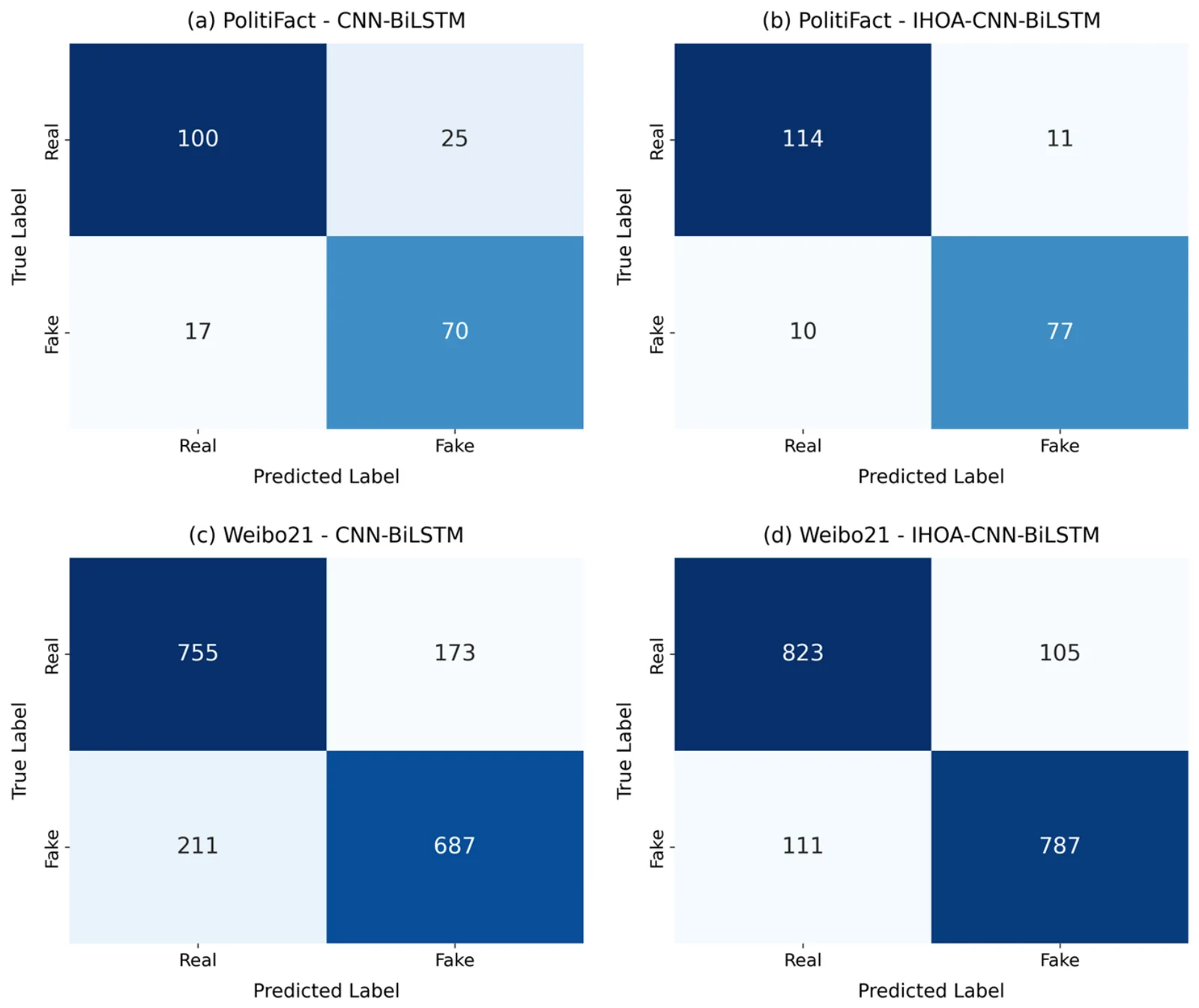
Confusion matrices on the PolitiFact and Weibo21 datasets. **Note:** The confusion matrix values are rounded integers from a representative run. Thus, minor rounding differences (within ±0.1%) from the averaged percentage metrics in Table 2 may occur.

To visually compare the classification performance of the CNN-BiLSTM model before and after hyperparameter optimization for fake information detection, this experiment presents the confusion matrices for both the unoptimized CNN-BiLSTM model and the IHOA-optimized model on the test set of the PolitiFact and Weibo21 datasets, as shown in Figure 4.

Figure 4 shows that the unoptimized CNN-BiLSTM model misclassifies a considerable number of fake-information samples as real. This issue may arise from inadequate capture of typical semantic patterns in fake information, due to inappropriate hyperparameter settings. In contrast, the IHOA-optimized model significantly reduces misclassifications across both categories, particularly false-negative predictions, indicating that the optimal hyperparameter combination fully leverages the dual-channel feature-extraction capability of the CNN-BiLSTM model. The same overall trend is observed across both datasets, indicating that IHOA-based hyperparameter optimization consistently improves the classification performance of the CNN-BiLSTM model under different dataset conditions. As a result, it effectively reduces missed detections while maintaining high precision. The visual analysis of the confusion matrices confirms the substantial improvement in detection performance achieved by IHOA optimization from a classification perspective.

#### 4.5.2. t-SNE Feature Visualization

**Figure 5:**
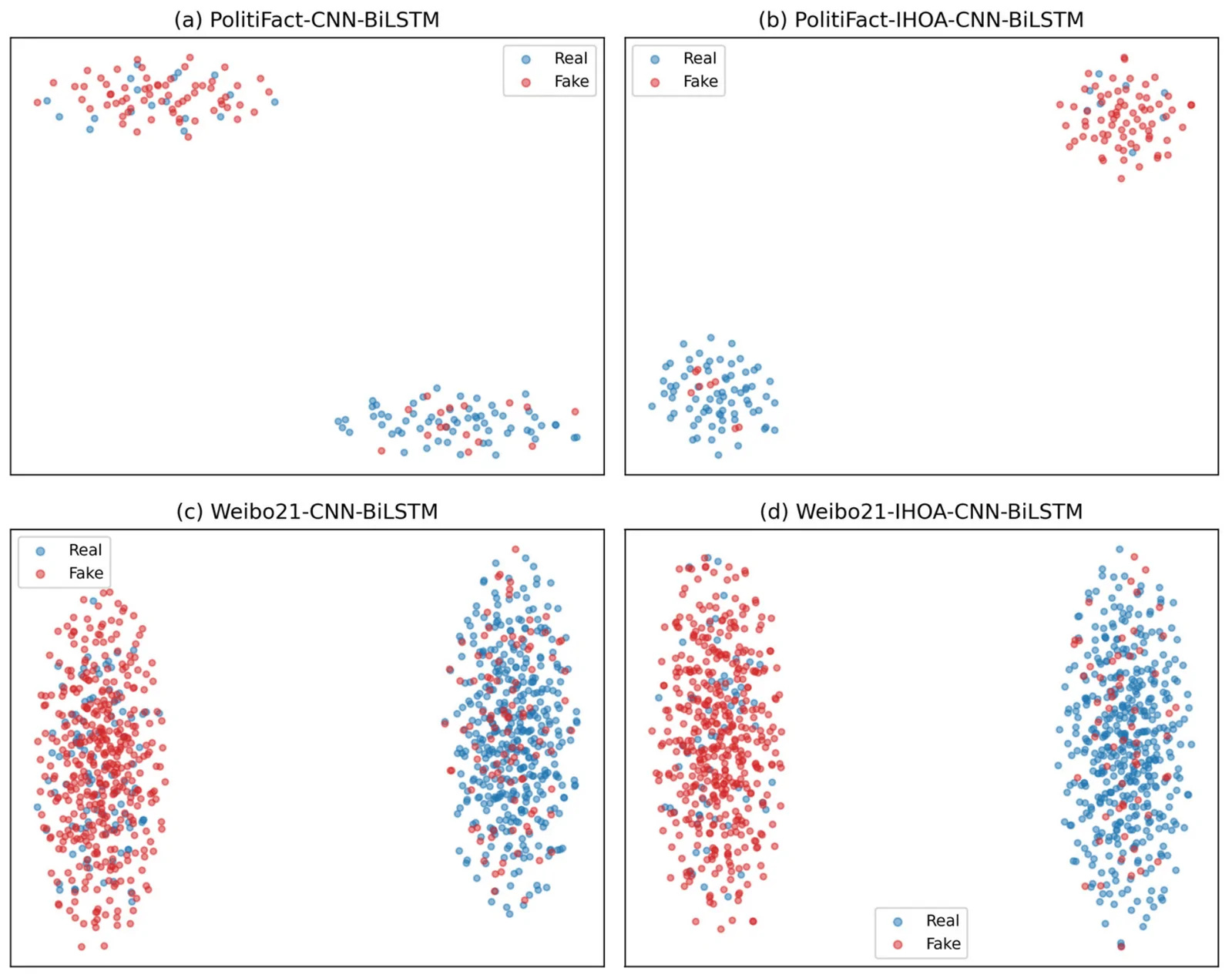
t-SNE visualization results on the PolitiFact and Weibo21 datasets.

To clearly illustrate the effect of hyperparameter optimization on the semantic extraction quality of the CNN-BiLSTM model, this experiment reduces the fused feature vectors extracted from test-set samples to a 2D space for visualization. The experiment is conducted on the PolitiFact and Weibo21 datasets, with comparison results displayed in Figure 5.

Figure 5a presents the feature distribution of the unoptimized CNN-BiLSTM model. There is significant overlap between the real and fake information samples in the two-dimensional space, leading to indistinct boundaries. A considerable number of fake information samples are embedded within the region of real information, and vice versa. This result is consistent with the model’s relatively low classification performance, indicating that it fails to learn discriminative semantic representations with the default hyperparameters and struggles to distinguish among features.

Figure 5b presents the feature distribution of the model optimized using IHOA. Samples of the same category are clustered more tightly, thereby enhancing separation between categories and clarifying the decision boundary. Meanwhile, a small number of samples from the two categories remain intermingled, consistent with the classification errors observed on the test set. Compared with Figure 5a, the optimized model exhibits more compact intra-class distributions and clearer inter-class boundaries on the PolitiFact dataset, indicating improved feature discrimination after hyperparameter optimization.

Figure 5c presents the feature distribution of the unoptimized CNN-BiLSTM model on the Weibo21 dataset. Similar to the results on PolitiFact, the real and fake information samples show considerable overlap, suggesting that the unoptimized model has limited ability to distinguish between the two classes. Some samples are distributed across the regions dominated by the opposite class, reflecting the classification ambiguity of the original feature representations.

Figure 5d shows the feature distribution of the IHOA-optimized CNN-BiLSTM model on Weibo21. Compared with Figure 5c, the two categories form more distinct clusters, with reduced overlap between real and fake information samples. Although a small number of boundary samples remain mixed, the overall feature distribution demonstrates improved inter-class separability and intra-class compactness. This result is consistent with the improved classification performance observed on the Weibo21 test set.

## 5. Conclusions and Future Work

### 5.1. Conclusions

This paper proposes an improved Hippopotamus Optimization Algorithm (IHOA) integrated with a trend-aware mechanism. It combines with a CNN-BiLSTM dual-channel hybrid deep learning model to construct the IHOA-CNN-BiLSTM framework for fake information detection. This framework significantly improves the accuracy and stability of fake information detection by enhancing population initialization and introducing intelligent guidance based on search trends.

Firstly, a Logistic-Tent hybrid chaotic mapping is used to reconstruct the population initialization process, effectively addressing the uneven distribution problem caused by traditional random initialization. This hybrid mapping combines the chaotic ergodicity of the Logistic mapping with the uniform distribution of the Tent mapping. Through dynamically adjusting adaptive weights, the initial population achieves broader coverage and more balanced distribution in the solution space. An ablation experiment confirms that the hybrid chaotic initialization yields a higher-quality set of starting points for the subsequent search by improving the distribution of the initial population.

Secondly, a trend-aware mechanism (TAM) is introduced to systematically record and analyze the population’s historical search positions and fitness information, thereby quantifying the underlying trends in the search process. TAM is embedded synchronously across the three search stages of HOA. It constructs trend vectors from historical trajectories and generates offset vectors with both directionality and randomness via an adaptive covariance mechanism, thereby providing intelligent guidance for candidate positions. Ablation experiment results confirm that TAM makes independent, superimposable positive contributions to model performance. Additionally, its synergistic effect with the hybrid chaotic mapping results in a final hyperparameter optimization that is significantly superior to those of single improvements or unimproved baseline configurations.

Thirdly, this paper constructs a parallel dual-channel CNN-BiLSTM feature-extraction network and employs the IHOA to optimize its hyperparameters. Multi-scale convolution operations are introduced in the CNN branch to extract fine-grained abnormal features from textual sequences, particularly irregular modifier combinations and atypical reference structures. Meanwhile, the BiLSTM branch exploits bidirectional sequence modeling to characterize dependencies across distant contexts and detect semantic inconsistencies, including broken causal relations and shifts in conceptual meaning. Their joint operation enables the model to integrate local structural cues with broader contextual semantics.

Finally, among the CNN-BiLSTM-based hybrid models, the proposed model achieves superior performance on both datasets, demonstrating its generalization capability. The analysis of the convergence curve, the comparison of the confusion matrix, and the visualization of features using t-SNE provide clear support for the experimental results across three dimensions: optimization efficiency, classification behavior, and representation learning.

### 5.2. Limitations and Future Work

While the IHOA-CNN-BiLSTM framework demonstrates strong performance in fake information detection, several limitations should be acknowledged. First, the model still has limited discriminative capability when dealing with texts containing highly ambiguous expressions, implicit factual conflicts, or complex narrative strategies. Although the parallel dual-channel architecture captures local anomalous patterns and long-range contextual dependencies, it does not explicitly incorporate external factual knowledge or evidence-based verification, which restricts its ability to identify subtle semantic contradictions and implicit logical fallacies.

Second, the scale and coverage of the datasets used in this study remain limited. The experiments are conducted on PolitiFact and Weibo21, which provide benchmarks in different linguistic and platform contexts but cannot fully represent the diversity and rapid evolution of real-world fake information across languages, domains, platforms, and emerging events. Consequently, the generalization capability of the proposed framework requires further validation on larger and more heterogeneous datasets.

Third, the metaheuristic hyperparameter optimization process introduces non-negligible computational costs. Each candidate solution generated by IHOA requires training and evaluating a CNN-BiLSTM model, and these repeated fitness evaluations are performed across multiple individuals and iterations. As discussed in the time-complexity analysis, the additional algorithmic overhead introduced by IHOA is relatively small compared with model training; nevertheless, the overall hyperparameter search remains computationally demanding, particularly as the dataset size, population size, number of iterations, or search space dimensionality increases.

Finally, the current implementation depends on the MATLAB 2024b environment. Although the source code and data are publicly available on GitHub, this software dependency may limit direct reproducibility for researchers outside the MATLAB ecosystem and introduce implementation differences when the framework is ported to other deep learning platforms.

Future work will address these limitations from several directions. External knowledge graphs and fact-verification modules will be incorporated to strengthen evidence-aware semantic reasoning. The framework will also be evaluated on larger, multilingual, and cross-platform datasets to examine its robustness and generalization under more diverse real-world conditions. In addition, more resource-efficient hyperparameter optimization strategies, such as early stopping, reduced search spaces, and surrogate-assisted evaluation, will be investigated to reduce the computational burden of repeated fitness evaluations. Cross-platform implementations based on widely used open-source deep learning frameworks will also be developed to improve the accessibility and reproducibility of the proposed method.

## Figures and Tables

**Table 1:** Experimental configuration settings.

Module/Process	Configuration Name	Value/Range	Description
IHOA	Population Size *N*	50	Number of individuals in the initial population
	Maximum Iterations *T*	100	Stopping criterion for hyperparameter tuning
	TAM Activation Threshold *n*	5	Minimum historical algebra TA required
	Recent Points *k*	3	Number of TA points
CNN-BiLSTM	Input Sequence Length *L*	256	Texts padded/truncated to uniform length
	Number of Conv Kernels *N_conv_*	{32, 64, 128}	Number of kernels per convolutional layer
	Convolution Kernel Size *H*	{2, 3, 4}, {2, 3, 5}, {3, 4, 5}	Three combinations of window sizes
	BiLSTM Hidden Units *N_lstm_*	{64, 128, 256}	Bidirectional LSTM unit count
	Number of Neurons in FC Layer *N_fc_*	{64, 128, 256}	Dimension of the FC layer after feature fusion
	Dropout Rate	[0.2, 0.6]	Uniform sampling from interval
Training and Testing	Optimizer	Adam	-
	Learning Rate *η*	[1 × 10^−4^, 1 × 10^−2^]	Log-uniform sampling
	L2 Regularization *λ*	[1 × 10^−5^, 1 × 10^−3^]	Log-uniform sampling
	Batch Size *B*	{32, 64, 128}	Number of samples per batch
	Epochs	100	Early stopping enabled (patience = 10)

**Table 2:** Results of the comparative experiments.

Models	PolitiFact Dataset					Weibo21 Dataset				
	Acc (%)	Pre (%)	Rec (%)	F1 (%)	*p*-Value	Acc (%)	Pre (%)	Rec (%)	F1 (%)	*p*-Value
CNN-BiLSTM	80.11 ± 1.16	73.60 ± 1.60	80.42 ± 2.19	76.84 ± 1.36	<0.001	78.99 ± 0.53	79.92 ± 0.63	76.51 ± 0.84	78.18 ± 0.58	<0.001
Random Search-CNN-BiLSTM	83.93 ± 1.91	79.81 ± 2.69	81.49 ± 4.22	80.62 ± 2.47	<0.001	81.22 ± 0.86	80.88 ± 1.01	80.96 ± 1.33	80.91 ± 0.90	<0.001
Bayesian Optimization-CNN-BiLSTM	85.38 ± 2.51	81.24 ± 3.76	83.87 ± 4.03	82.48 ± 3.00	<0.001	83.02 ± 0.71	82.52 ± 0.82	83.07 ± 1.09	82.79 ± 0.75	<0.001
TPE-CNN-BiLSTM	86.32 ± 2.05	82.31 ± 2.98	85.02 ± 4.11	83.60 ± 2.57	<0.001	84.28 ± 0.75	83.84 ± 0.82	84.29 ± 1.24	84.06 ± 0.81	<0.001
Hyperband-CNN-BiLSTM	87.74 ± 1.74	84.32 ± 2.50	86.25 ± 3.85	85.21 ± 2.23	<0.001	85.98 ± 0.65	85.51 ± 0.80	86.08 ± 0.95	85.79 ± 0.67	<0.001
GWO-CNN-BiLSTM	83.46 ± 2.51	78.31 ± 3.05	82.68 ± 4.81	80.37 ± 3.11	<0.001	81.38 ± 0.72	82.23 ± 0.92	79.28 ± 1.06	80.72 ± 0.76	<0.001
WOA-CNN-BiLSTM	84.47 ± 2.51	79.47 ± 3.10	83.91 ± 4.48	81.57 ± 3.08	<0.001	81.38 ± 0.68	82.53 ± 0.83	78.84 ± 1.23	80.63 ± 0.77	<0.001
BWO-CNN-BiLSTM	85.44 ± 2.43	81.24 ± 3.22	83.98 ± 3.57	82.56 ± 2.93	<0.001	80.50 ± 0.53	81.37 ± 0.68	78.29 ± 0.94	79.79 ± 0.59	<0.001
BKA-CNN-BiLSTM	86.38 ± 2.25	82.41 ± 3.42	85.13 ± 3.57	83.69 ± 2.67	<0.001	82.37 ± 0.74	83.41 ± 0.84	80.07 ± 1.00	81.70 ± 0.78	<0.001
HOA-CNN-BiLSTM	87.77 ± 1.57	84.38 ± 2.64	86.28 ± 3.22	85.26 ± 1.94	<0.001	83.18 ± 0.62	84.32 ± 0.78	80.84 ± 0.74	82.54 ± 0.64	<0.001
BERT	88.25 ± 1.70	85.30 ± 2.53	86.32 ± 2.80	85.78 ± 2.06	<0.010	85.43 ± 0.42	84.80 ± 0.54	85.74 ± 0.75	85.26 ± 0.44	<0.001
RoBERTa	91.02 ± 1.20	88.68 ± 1.97	89.62 ± 2.61	89.11 ± 1.49	0.070	86.37 ± 0.63	86.19 ± 0.50	86.08 ± 0.61	86.13 ± 0.37	<0.001
IHOA-CNN-BiLSTM	90.13 ± 2.15	87.62 ± 3.08	88.54 ± 3.81	88.02 ± 2.64	—	88.18 ± 0.60	88.26 ± 0.86	87.62 ± 0.72	87.94 ± 0.60	—

**Table 3:** Computational complexity comparison of different algorithms.

Models	Accuracy (%)	F1-Score (%)	Params (M)	FLOPs (G)
GWO-CNN-BiLSTM	81.38 ± 0.72	80.72 ± 0.76	12.8	25.6
WOA-CNN-BiLSTM	81.38 ± 0.68	80.63 ± 0.77	13.1	26.2
BWO-CNN-BiLSTM	80.50 ± 0.53	79.79 ± 0.59	13.6	27.2
BKA-CNN-BiLSTM	82.37 ± 0.74	81.70 ± 0.78	14.0	28.0
HOA-CNN-BiLSTM	83.18 ± 0.62	82.54 ± 0.64	14.2	28.5
IHOA-CNN-BiLSTM	88.18 ± 0.60	87.94 ± 0.60	15.0	30.1

**Table 4:** Ablation experiments result on the Weibo21 dataset.

Models	Accuracy (%)	Precision (%)	Recall (%)	F1-Score (%)	*p*-Value
w/o TAM and LT	83.71 ± 0.86	83.95 ± 1.10	83.02 ± 1.04	83.48 ± 0.96	<0.001
w/o TAM	84.45 ± 0.73	84.23 ± 0.94	84.06 ± 0.88	84.14 ± 0.81	<0.001
w/o LT	85.19 ± 0.67	85.08 ± 0.88	84.96 ± 0.76	85.02 ± 0.73	<0.001
**IHOA-CNN-BiLSTM**	**88.18 ± 0.60**	**88.26 ± 0.86**	**87.62 ± 0.72**	**87.94 ± 0.60**	—

## Data Availability

The original code and data are openly available in GitHub at https://github.com/IHOA-CNN-BiLSTM/IHOA-CNN-BiLSTM (accessed on 6 July 2026).
